# Aetiology of Acute Respiratory Tract Infections in Hospitalised Children in Cyprus

**DOI:** 10.1371/journal.pone.0147041

**Published:** 2016-01-13

**Authors:** Jan Richter, Christakis Panayiotou, Christina Tryfonos, Dana Koptides, Maria Koliou, Nikolas Kalogirou, Eleni Georgiou, Christina Christodoulou

**Affiliations:** 1 Cyprus Institute of Neurology and Genetics, Department of Molecular Virology, Nicosia, Cyprus; 2 Archbishop Makarios III Hospital, Department of Pediatrics, Nicosia, Cyprus; Nanyang Technical University, UNITED STATES

## Abstract

In order to improve clinical management and prevention of viral infections in hospitalised children improved etiological insight is needed. The aim of the present study was to assess the spectrum of respiratory viral pathogens in children admitted to hospital with acute respiratory tract infections in Cyprus. For this purpose nasopharyngeal swab samples from 424 children less than 12 years of age with acute respiratory tract infections were collected over three epidemic seasons and were analysed for the presence of the most common 15 respiratory viruses. A viral pathogen was identified in 86% of the samples, with multiple infections being observed in almost 20% of the samples. The most frequently detected viruses were RSV (30.4%) and Rhinovirus (27.4%). RSV exhibited a clear seasonality with marked peaks in January/February, while rhinovirus infections did not exhibit a pronounced seasonality being detected almost throughout the year. While RSV and PIV3 incidence decreased significantly with age, the opposite was observed for influenza A and B as well as adenovirus infections. The data presented expand our understanding of the epidemiology of viral respiratory tract infections in Cypriot children and will be helpful to the clinicians and researchers interested in the treatment and control of viral respiratory tract infections.

## Introduction

Viral Respiratory tract infections (RTI) represent a major public health problem because of their world-wide occurrence, ease of transmission and considerable morbidity and mortality effecting people of all ages. Children are on average infected two to three times more frequently than adults, with acute RTIs being the most common infection in childhood [1,2]. Illnesses caused by respiratory viruses include, among others, common colds, pharyngitis, croup, bronchiolitis, viral pneumonia and otitis media. Rapid diagnosis is important not only for timely therapeutic intervention but also for the identification of a beginning influenza epidemic and the avoidance of unnecessary antibiotic treatment [3,4].

RTIs are a major cause of morbidity and mortality worldwide. Acute RTI is most common in children under five years of age, and represents 30–50% of the paediatric medical admissions, as well as 20–40% of hospitalizations in children. Respiratory infections cluster during winter and early spring months. The leading viral agents include respiratory syncytial virus (RSV), influenza A and B (INF-A, INF-B) viruses, parainfluenza viruses (PIVs), and human adenoviruses (HAdVs). In addition, there is a continuously increasing list of new respiratory viruses that contribute significantly to the burden of acute respiratory infections, such as the recently identified human metapneumovirus (HMPV) and human Bocavirus (HBoV) [5].

Acute RTIs are classified as upper (UTRIs) and lower RTI (LRTIs), according to the involved anatomic localization. URTIs cause non-severe but widespread epidemics that are responsible for continuous circulation of pathogens in the community. LRTIs have been classified as frank pneumonia and bronchiolitis with clinical, radiological and etiological features that usually overlap [6,7]. Viruses are again the foremost agents of LRTIs often misdiagnosed as bacterial in origin and hence treated with antibiotics unnecessarily [8].

The main aim of this study was to determine the aetiology of acute respiratory tract infections in Cypriot children and assess the epidemiology of the identified viral pathogens over three epidemic seasons.

## Materials and Methods

### Patients and clinical specimens

The study was approved by the Cyprus National Bioethics Committee. Accordingly, written informed consent was obtained from parents prior to sample taking. Between November 2010 and October 2013, 485 nasopharyngeal swab samples were collected from children up to 12 years of age, who had been hospitalized with acute respiratory tract infection at the Archbishop Makarios III hospital, Nicosia. Clinical and demographic information including symptoms, duration of hospitalisation, diagnosis and treatment were recorded. Nasal swab samples were collected using the BD Universal Viral Transport Collection Kit. Viral RNA/DNA was extracted from 400 μl sample using the iPrep PureLink Virus Kit on an iPrep purification instrument (Invitrogen).

### Virus detection

A set of four multiplex Real-Time RT-PCR assays was established and validated for the detection of the 15 most common respiratory viruses as follows: assay 1: influenzaviruses A and B, RSV, assay 2: parainfluenzaviruses 1–4, assay 3: HAdV, enteroviruses, HMPV and HBoV and assay 4: rhinoviruses and the human coronaviruses OC43, NL63 and 229E (Table 1).

**Table 1:** Primers and probes used in four Multiplex-assays for the detection of 15 different viral pathogens.

	Virus	Probe label	Gene targeted	Reference	Comments
**Assay 1**	Inf A	FAM	Matrix protein 2	CDC H1N1[9]	
	Inf B	Yakima Yellow	Nuclear export protein	Selvajaru et al. [10]	
	RSV	TAMRA	Matrix protein	Fry et al. [11]	Probe modified*
**Assay 2**	PIV 1	FAM	HN	Watzinger et al. [12]	
	PIV 2	JOE	HN	Watzinger et al. [12]	
	PIV 3	TAMRA	HN	Watzinger et al. [12]	
	PIV 4	Cy5	Phosphoprotein	Templeton et al. [13]	Probe modified*
**Assay 3**	HCoV OC43	FAM	Nucleocapsid protein	Tiveljung et al. [14]	
	HCoV NL63	Yakima Yellow	Nucleocapsid protein	Tiveljung et al. [14]	
	HCoV 229E	TAMRA	Nucleocapsid protein	Tiveljung et al. [14]	
	Rhinovirus	Cy5	5’UTR	Lu et al. [15]	Additional F primer*
**Assay 4**	Enterovirus	FAM	5’UTR	Tryfonos et al. [16]	
	HBoV	Yakima Yellow	Nonstructural protein	Tiveljung et al. [14]	
	HAdV	TAMRA	Hexon protein	Heim et al. [17]	Probe modified*
	HMPV	Cy5	Nucleoprotein	Tiveljung [14]	

^*^The sequences of the modified primers/probes are shown in supplements S1–S4 Files.

Published primer and probe sets were used as a basis for designing the assays, however, all primer/probe sequences were checked against newly build sequence alignments of all viruses tested and were modified, if necessary, to account for possible sequence variations. For this purpose, all available complete genome sequences were obtained for each virus from GenBank, imported into the BioEdit Sequence Alignment Editor v7.1.7 and aligned using ClustalX. In case of mismatches between published primers/probe and target sequences, modifications were applied, as indicated in Table 1. The alignments for the viruses, which necessitated changes to the primers/probe are available in Fasta-Format as supplement S1–S4 Files.

Primer concentrations and reaction conditions for the four assays were subsequently optimised for multiplexing. In order to assess the sensitivity and specificity of the assays, the laboratory enrolled for two consecutive years in Quality Control for Molecular Diagnostics (QCMD) external quality assessment schemes for all viruses, except Bocavirus, which was unavailable. In summary, the established assays were able to correctly identify all viruses tested, proving their suitability for diagnostic application.

### Statistical Analysis

A possible correlation of virus prevalence and age of infection was assessed using univariate analyses. The Fisher’s exact test was used where cell counts below 5 were encountered; otherwise, the chi-squared test was performed. The same statistical tests were used to compare the frequency of subjects with single or multiple infections between age groups. In addition, Pearson correlation was used to examine co-infections of different viruses. All statistical analyses were performed using StataSE 12 (StatCorp. 2007. College Station, TX, USA).

## Results

**Fig 1:**
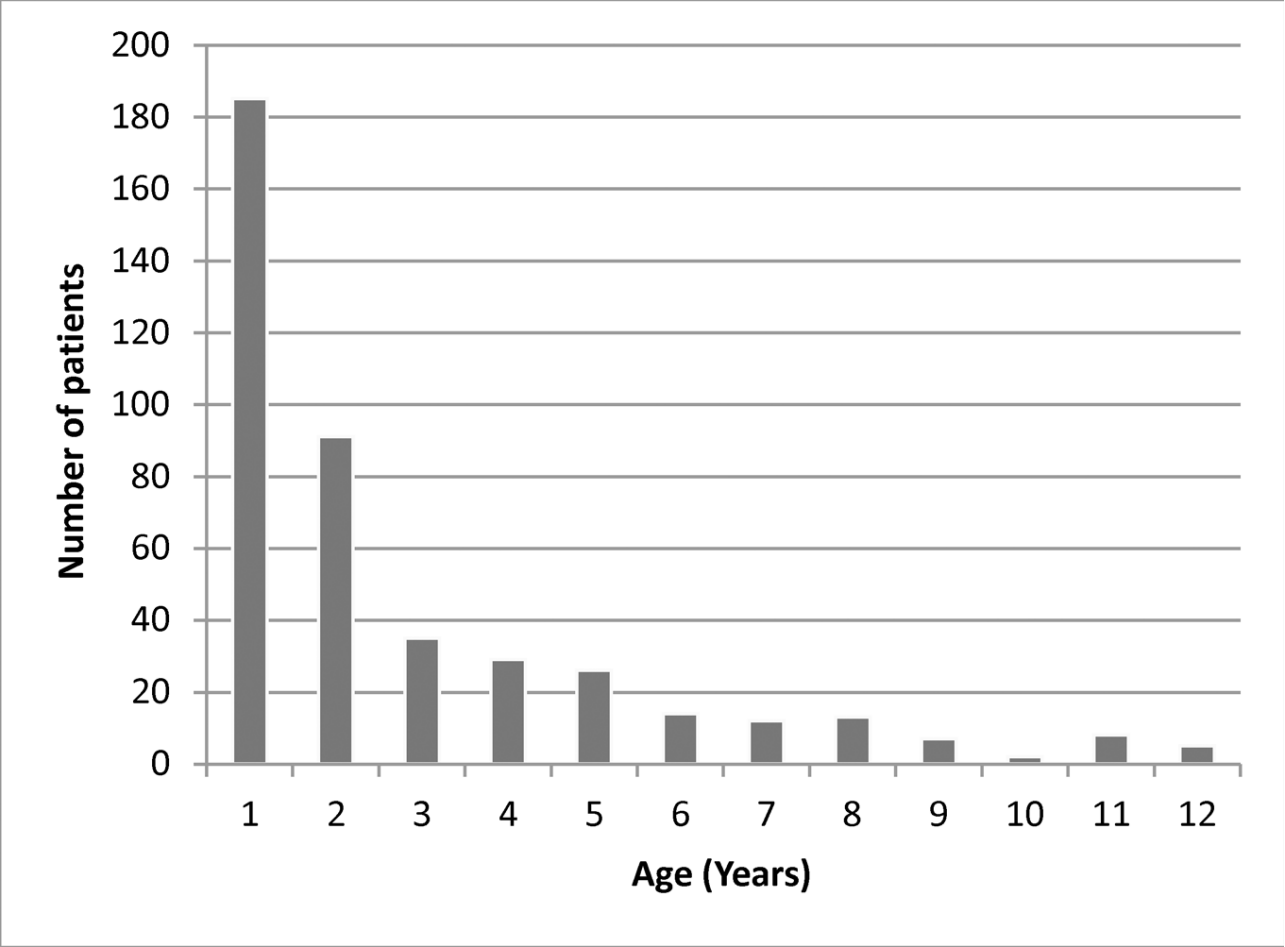
Age distribution of paediatric patients hospitalised with acute respiratory infections.

The present study was a prospective investigation of children hospitalized with acute respiratory tract infections between November 2010 and October 2013 in Cyprus. The median age of the children was 15 months (range: 0–140 months) with 243 being male and 181 female (male/female ratio 1.34). The age distribution is shown in Fig 1.

### Viral aetiologies

Out of the 424 samples analysed, 364 (85.8%) were positive for one or more viruses. Results are summarized in Table 2.The most commonly detected viruses were RSV, which was found in 129 (30.4%) patients and rhinoviruses in 116 (27.4%) accounting together for almost 60% of all detections. With moderate frequency have been detected HAdV in 31(7.3%) patients, influenza A in 28 (6.6%), HBoV in 24 (5.7%), enteroviruses and PIV 3 in 23 (5.4%) of patients respectively, and Influenza B in 21 (5.0%). A low frequency was exhibited by HMPV with 16 (3.8%) positive samples, human coronavirus OC43 with 13 (3.1%), PIV 1 with 12 (2.8%), PIV 4 with 9 (2.1%), PIV 2 with 7 (1.7%) and HCoV NL63 with 6 (1.4%). Coronavirus 229E could be detected only in a single sample.

**Table 2:** Distribution of virus infection by patient age.

Virus	0–3 months,n = 74		4–12 months,n = 108		13–36 months,n = 127		3–12 years,n = 115		Total,n = 424		P-value^α^
**RSV**	**43**	58.1%	**41**	38.0%	**30**	23.6%	**15**	13.0%	**129**	30.4%	**<0.0001**
**HRV**	**15**	20.3%	**33**	30.6%	**42**	33.1%	**26**	22.6%	**116**	27.4%	0.121
**HAdV**	**0**	0.0%	**11**	10.2%	**14**	11.0%	**6**	5.2%	**31**	7.3%	**0.005**
**Inf A**	**2**	2.7%	**3**	2.8%	**9**	7.1%	**14**	12.2%	**28**	6.6%	**0.021**
**HBoV**	**1**	1.4%	**12**	11.1%	**9**	7.1%	**2**	1.7%	**24**	5.7%	**0.006**
**HEV**	**2**	2.7%	**7**	6.5%	**10**	7.9%	**4**	3.5%	**23**	5.4%	**0.033**
**PIV 3**	**6**	8.1%	**7**	6.5%	**7**	5.5%	**3**	2.6%	**23**	5.4%	0.339
**Inf B**	**0**	0.0%	**2**	1.9%	**4**	3.1%	**15**	13.0%	**21**	5.0%	**<0.0001**
**MPV**	**3**	4.1%	**3**	2.8%	**8**	6.3%	**2**	1.7%	**16**	3.8%	0.298
**HCoV OC43**	**1**	1.4%	**4**	3.7%	**2**	1.6%	**6**	5.2%	**13**	3.1%	0.365
**PIV 1**	**1**	1.4%	**3**	2.8%	**5**	3.9%	**3**	2.6%	**12**	2.8%	0.816
**PIV 4**	**3**	4.1%	**1**	0.9%	**1**	0.8%	**4**	3.5%	**9**	2.1%	0.249
**PIV 2**	**0**	0.0%	**2**	1.9%	**3**	2.4%	**2**	1.7%	**7**	1.7%	0.695
**HCoV NL63**	**2**	2.7%	**0**	0.0%	**3**	2.4%	**1**	0.9%	**6**	1.4%	0.671
**HCoV 229E**	**0**	0.0%	**0**	0.0%	**0**	0.0%	**1**	0.9%	**1**	0.2%	0.700
** **											
**Negative**	**7**	9.5%	**9**	8.3%	**19**	15.0%	**25**	21.7%	**60**	14.2%	**0.020**
**Multiple infection**	**11**	14.9%	**27**	25.0%	**32**	25.2%	**13**	11.3%	**83**	19.6%	**0.014**

^a^ The correlation of virus prevalence and age was assessed using univariate analyses. P-values were calculated using the chi-squared test or Fisher’s exact test, where cell counts below 5 were observed. P-values less than 0.05 are indicated in bold.

### Multiple Infections

Co-infections with two or more viruses were observed in 84 out of the 364 positive samples (see Table 2). Dual infections accounted for 17% of all positive samples and three viruses were detected in 2.7% of samples). A single patient sample displayed a quadruple infection being simultaneously positive for RSV, rhinovirus, HBoV and influenza B. Table 3 summarizes the frequency of each virus in single vs. multiple infections as well as the number of co-occurrences of viruses for each possible virus combination. In absolute terms the most common combination observed was RSV/rhinovirus. As a percentage, however, the virus appearing most often in co-infections was HBoV, which was found in more than 70% of cases together with another virus, followed by coronaviruses HCoV OC43 and HCoV NL63 with 61% and 67%, respectively. On the other hand, the viruses most rarely seen in co-infections were influenza viruses A and B as well as RSV. Pearson correlation coefficients were calculated to examine the likelihood of co-infections of different viruses. The results of the analysis are summarized in Table 1 in S1 Table. Significant correlation (P-value < 0.05) was seen mostly for co-infections with RSV, however correlations were very weak (r<0.3) and negative. This finding can probably be explained by the fact that RSV infections occurred predominantly in the very young, where co-infections were less frequently observed. On the other hand, a significant positive correlation was observed for enterovirus and rhinovirus co-infection hinting maybe at similarities in circulation patterns and/or transmission modes.

**Table 3:** Rates of detection of 15 viral pathogens in children hospitalized with acute RTI (Nov. 2010—Oct. 2013) and occurrence of multiple infections.

	Total	Single	Double	Triple	Quad	Mixed infection	RSV	HRV	HAdV	Inf A	HBoV	HEV	PIV3	Inf B	MPV	HCoV OC43	PIV1	PIV4	PIV2	HCoV NL63	HCoV 229E
**RSV**	**129**	**94**	**28**	**6**	**1**	**27.1%**		17	2	2	9	2	2	2	1	3	1	2	0	0	0
**HRV**	**116**	**62**	**46**	**7**	**1**	**46.6%**	17		6	2	7	12	4	3	2	3	4	1	2	0	0
**HAdV**	**31**	**19**	**9**	**3**	**0**	**38.7%**	2	6		1	1	0	0	0	1	2	0	0	1	0	1
**Inf A**	**28**	**21**	**7**	**0**	**0**	**25.0%**	2	2	1		0	0	0	0	0	1	0	0	0	1	0
**HBoV**	**24**	**7**	**12**	**4**	**1**	**70.8%**	9	7	1	0		1	0	1	0	1	0	1	1	1	0
**HEV**	**23**	**10**	**9**	**4**	**0**	**56.5%**	2	12	0	0	1		1	0	0	0	1	0	0	0	0
**PIV3**	**23**	**17**	**5**	**1**	**0**	**26.1%**	2	4	0	0	0	1		0	0	0	0	0	0	0	0
**Inf B**	**21**	**17**	**3**	**0**	**1**	**19.0%**	2	3	0	0	1	0	0		0	0	0	0	0	0	0
**MPV**	**16**	**10**	**6**	**0**	**0**	**37.5%**	1	2	1	0	0	0	0	0		0	0	0	0	2	0
**HCoV OC43**	**13**	**5**	**6**	**2**	**0**	**61.5%**	3	3	2	1	1	0	0	0	0		0	0	0	0	0
**PIV1**	**12**	**7**	**4**	**1**	**0**	**41.7%**	1	4	0	0	0	1	0	0	0	0		0	0	0	0
**PIV4**	**9**	**6**	**2**	**1**	**0**	**33.3%**	2	1	0	0	1	0	0	0	0	0	0		0	0	0
**PIV2**	**7**	**4**	**2**	**1**	**0**	**42.9%**	0	2	1	0	1	0	0	0	0	0	0	0		0	0
**HCoV NL63**	**6**	**2**	**4**	**0**	**0**	**66.7%**	0	0	0	1	1	0	0	0	2	0	0	0	0		0
**HCoV 229E**	**1**	**0**	**1**	**0**	**0**	**100.0%**	0	0	1	0	0	0	0	0	0	0	0	0	0	0	

### Seasonality

**Fig 2:**
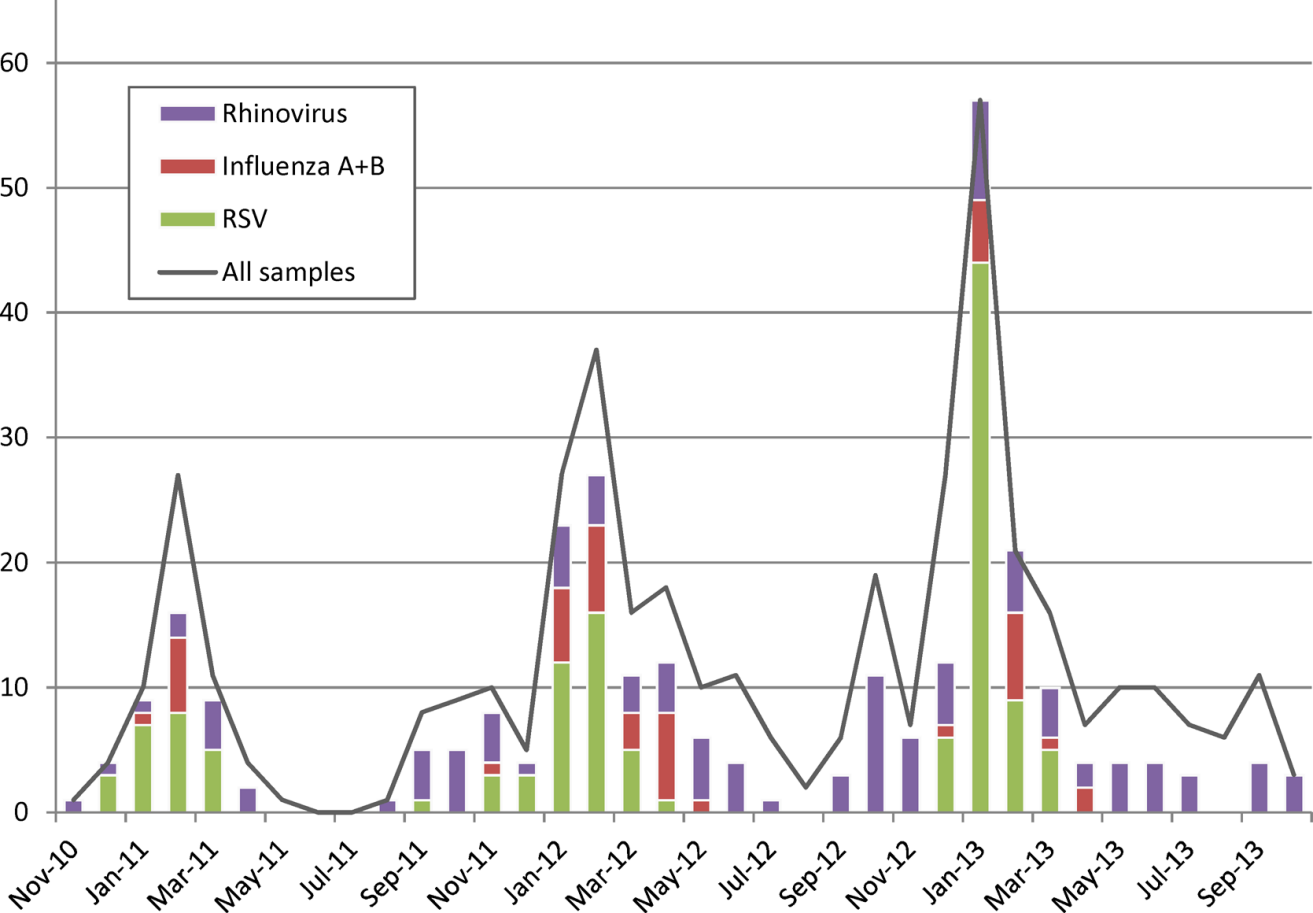
Seasonality of RSV, rhinovirus and influenza virus infections.

Regarding seasonality, different patterns of circulations could be observed for RSV, rhinoviruses and influenzaviruses (A and B combined) (Fig 2), with RSV and influenza exhibiting a clear seasonality with marked peaks in January/February, while rhinovirus infections did not exhibit a pronounced seasonality being detected almost throughout the year. However, as more than 100 different rhinovirus strains have been identified to be circulating worldwide in parallel and successively, a potential seasonality of individual rhinovirus serotypes may be masked by overlapping patterns [18,19].

### Age group characteristics of viral infection

**Fig 3:**
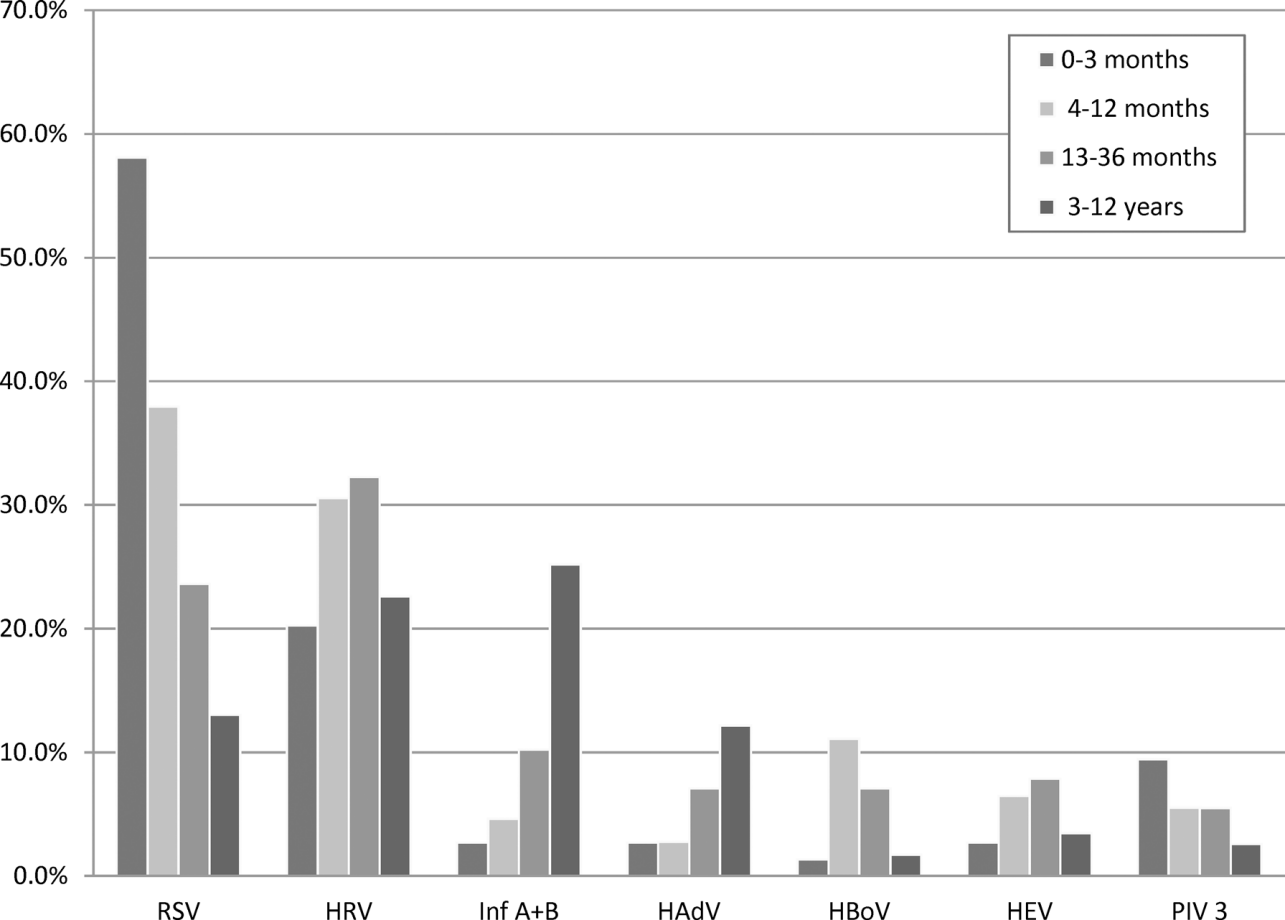
Distribution of virus infection by patient age for the 7 most frequently detected viruses.

The data was further analysed with regard to the age distribution of virus infection (see Table 2). In infants up to 3 months old, RSV was by far the most common pathogen (58.1%), followed by rhinovirus (20.3%) and PIV3 with 8.1% each. The incidence of RSV, however, decreases significantly with increasing age (p-value < 0.0001) dropping to 13% in children older than 3 years old, while the reverse relationship is observed for Influenza A and B and HAdV. Rhinoviruses, HBoV and enteroviruses are most frequently observed in children from 4 months to 3 years of age. The age dependency of the virus incidence is visualized in Fig 3 for the seven most frequently observed viruses. The positivity rate also showed a trend according to the age group dropping from 90.5% in the under 3-month old to 78.3% in the 4–12 years old (p-value = 0.020). This may point to an increasing role of pathogens not included in the assays, such as bacterial infections in older children.

Regarding multiple infections, children less than 3 month of age and those older than 4 years had a significantly smaller risk to present with multiple infections as compared to the other two age groups (p-value = 0.014).

A reason for this could be that very young children have limited contact to others reducing thereby the chance for a co-infection, whereas children older than 3 years already established immunity to an increasing number of viruses encountered previously.

## Discussion

This study for the first time examined the aetiology of acute respiratory tract infections in hospitalised children in Cyprus. Four multiplex Real-Time RT-PCR assays were developed in order to detect the most common respiratory viral pathogens in a fast and cost-effective way. The high rate of positive samples (85.8%) is evidence of the high sensitivity of the Multiplex-assays used and that the range of viruses included in the analysis is comprehensive. Many previous studies have shown detection rates ranging from below 50% to 75% [20–24].

The most common viruses detected were RSV and rhinovirus accounting for almost 60% of all cases. Both viruses were reported previously by others as the major aetiology for respiratory viral infections in young children with rhinoviruses being recognized increasingly for their role in lower respiratory tract infections [20,25–30].

Our data support the results of similar studies performed in the Middle East region. A recently published study found that RSV was the most commonly detected virus in nasopharyngeal swabs from children presenting symptoms of RTIs and in addition to that it also showed that RSV infections follow a similar circulation pattern peaking from December to March [31]. Another study has revealed that RSV and PIV3 incidence decreases significantly with age, whereas the opposite is observed for influenza and adenovirus infections, a trend that was also observed in our study [26].

Mixed infections were observed in approximately 20% of all samples, which is in the middle of previously reported rates ranging from 10 to almost 40%. HBoV, HCoV and EV were found most frequently in co-infections. All three subtypes of HCoV were co-detected with several other viruses, while HBoV was co-detected mainly with HRV and RSV. In the case of EV infections, EV were almost predominantly associated with HRV. The rare presence of InfA and InfB viruses in multiple infections witnessed in our study was also observed elsewhere [32,33]. Even though this study did not allow for investigating a possible association between multiple infections and disease severity, a review of the literature shows that such a potential association is still subject to controversy, since there are reports showing no relationship of multiple virus infection with respiratoty illness severity on one hand or a significant association on the other. Studies have shown that viral co-infection was significantly associated with longer duration of illness symptoms, but with a decreased severity in hospitalized children regarding oxygen requirement and intensive care unit admission, whereas the findings of other studies have indicated that severe clinical phenotypes were more prevalent in co-infection patients, especially in RSV co-infections that may increase the severity of RSV associated disease in children [25,34–40].

Viral respiratory infections continue to be a worldwide health concern. As the clinical symptoms of patients with acute respiratory tract infections do usually not allow a discrimination of viral or bacterial aetiology, rapid and reliable diagnostic tools are required for better antibiotic stewardship and the implementation of appropriate infection control measures [4,41]. The data presented expand our understanding of the epidemiology of viral respiratory tract infections in Cypriot children and will be helpful to the clinicians and researchers interested in the treatment and control of viral respiratory tract infections.

## Supporting Information

S1 File Alignment of RSV sequences. (FAS)

S2 File Alignment of PIV 4 sequences. (FAS)

S3 File Alignment of rhinovirus sequences. (FAS)

S4 File Alignment of adenovirus sequences. (FAS)

S1 Table Pearson Correlation coefficients for observed viral co-infections. (PDF)
