# Supplementary material for: Aetiology of Acute Respiratory Tract Infections in Hospitalised Children in Cyprus
Source: PLoS One. 2016 Jan 13;11(1):e0147041. doi: 10.1371/journal.pone.0147041 (PMC4720120; doi:10.1371/journal.pone.0147041)
Supplement: S1 Table — (PDF) [file pone.0147041.s005.pdf]

S1 Table 1. Pearson Correlation coefficients for observed viral co-infections.

	rsv	hrv	hadv	infa	hbov	hev	piv3	infb	mpv	oc43	piv1	piv4	piv2	nl63	229E
**rsv**	**1.0000**														
**hrv**	**-0.2103**	**1.0000**													
	0.0000														
**hadv**	**-0.1463**	**-0.0504**	**1.0000**												
	0.0025	0.3003													
**infa**	**-0.1346**	**-0.1206**	**-0.0382**	**1.0000**											
	0.0055	0.0130	0.4327												
**hbov**	**0.0377**	**0.0099**	**-0.0296**	**-0.0651**	**1.0000**										
	0.4391	0.8384	0.5434	0.1807											
**hev**	**-0.1131**	**0.1333**	**-0.0673**	**-0.0637**	**-0.0136**	**1.0000**									
	0.0198	0.0060	0.1668	0.1906	0.7800										
**piv3**	**-0.1131**	**-0.0535**	**-0.0673**	**-0.0637**	**-0.0587**	**-0.0114**	**1.0000**								
	0.0198	0.2713	0.1668	0.1906	0.2280	0.8152									
**infb**	**-0.1037**	**-0.0669**	**-0.0641**	**-0.0607**	**-0.0089**	**-0.0547**	**-0.0547**	**1.0000**							
	0.0328	0.1689	0.1876	0.2123	0.8554	0.2613	0.2613								
**mpv**	**-0.1041**	**-0.0660**	**-0.0081**	**-0.0527**	**-0.0485**	**-0.0474**	**-0.0474**	**-0.0452**	**1.0000**						
	0.0322	0.1749	0.8683	0.2793	0.3190	0.3299	0.3299	0.3531							
**oc43**	**-0.0284**	**-0.0171**	**0.0552**	**0.0078**	**0.0156**	**-0.0426**	**-0.0426**	**-0.0406**	**-0.0352**	**1.0000**					
	0.5597	0.7258	0.2571	0.8728	0.7481	0.3817	0.3817	0.4044	0.4695						
**piv1**	**-0.0819**	**0.0229**	**-0.0479**	**-0.0454**	**-0.0418**	**0.0219**	**-0.0409**	**-0.0390**	**-0.0338**	**-0.0304**	**1.0000**				
	0.0920	0.6386	0.3248	0.3512	0.3905	0.6527	0.4012	0.4236	0.4876	0.5331					
**piv4**	**-0.0263**	**-0.0537**	**-0.0414**	**-0.0392**	**0.0347**	**-0.0353**	**-0.0353**	**-0.0336**	**-0.0292**	**-0.0262**	**-0.0251**	**1.0000**			
	0.5898	0.2702	0.3956	0.4212	0.4756	0.4689	0.4689	0.4900	0.5493	0.5907	0.6058				
**piv2**	**-0.0857**	**0.0035**	**0.0347**	**-0.0345**	**0.0484**	**-0.0310**	**-0.0310**	**-0.0296**	**-0.0257**	**-0.0230**	**-0.0221**	**-0.0191**	**1.0000**		
	0.0780	0.9423	0.4759	0.4792	0.3205	0.5240	0.5240	0.5436	0.5983	0.6361	0.6498	0.6952			
**nl63**	**-0.1176**	**-0.0478**	**0.0026**	**0.0078**	**0.0748**	**-0.0426**	**-0.0426**	**-0.0406**	**0.1084**	**-0.0316**	**-0.0304**	**-0.0262**	**0.7285**	**1.0000**	
	0.0154	0.3265	0.9574	0.8728	0.1239	0.3817	0.3817	0.4044	0.0257	0.5160	0.5331	0.5907	0.0000		
**229e**	**-0.0322**	**-0.0298**	**0.1731**	**-0.0129**	**-0.0119**	**-0.0116**	**-0.0116**	**-0.0111**	**-0.0096**	**-0.0086**	**-0.0083**	**-0.0072**	**-0.0063**	**-0.0086**	**1.0000**
	0.5091	0.5400	0.0003	0.7907	0.8068	0.8110	0.8110	0.8197	0.8433	0.8591	0.8647	0.8831	0.8971	0.8591	

Under the correlation coefficients, which are indicated in bold, the respective P-values are shown.
